# The impact of economic burden of disease on poverty among young migrant workers: evidence from China

**DOI:** 10.3389/fpubh.2025.1496014

**Published:** 2025-02-07

**Authors:** Yiyan Chen, Yueping Yan, Lijun Chen

**Affiliations:** ^1^School of Public Administration, Zhejiang University, Hangzhou, China; ^2^School of Ethnology and Sociology, Yunnan University, Kunming, China

**Keywords:** economic burden of disease, young migrant workers, multidimensional poverty, financial toxicity, gender differences

## Abstract

**Introduction:**

The treatment cost expenditure for family members with illness is a significant factor exacerbating poverty. Using nationally representative data concerning the migrant population, this study analyzes the impact of the economic burden from family members’ illness on poverty among young migrant workers.

**Methods:**

Firstly, we construct a multidimensional poverty index system based on five dimensions: income, education, employment, health, and livelihood,and used the A-F method for calculation. Moreover, diverging from existing studies that objectively measure the economic burden of disease, we explore an evaluation method based on subjective perceptions.

**Results:**

The incidence of multidimensional poverty among young migrant workers is relatively high, at 30.93%. The economic burden imposed by family members’ illnesses significantly exacerbates their risk of poverty, particularly within the 26-35 age bracket. Furthermore, compared to their male counterparts, female young migrant workers are more susceptible to the adverse effects of the economic burden of disease.

**Discussion:**

In China, migrant workers confront numerous challenges in both their professional and personal lives, with the economic burden on their families further exacerbating their predicament. Consequently, policymakers should prioritize enhancing health insurance coverage and expanding the reach of social welfare programs to alleviate poverty and the pressures faced by young migrant workers who are the primary breadwinners.

## 1 Introduction

The concept of Global Burden of Disease (GBD) was initially introduced by the World Bank in the World Development Report 1993: Investment and Health. Subsequently, GBD research has disseminated a series of scholarly analyses, annual assessments, and forecasts in various formats (1, 2), which have been extensively utilized by governments and various sectors of society. Under the framework of the United Nations Sustainable Development Goals (SDGs), an increasing number of countries are conducting localized assessments at the onset of each GBD cycle, providing a solid foundation for health policy interventions by estimating health losses with practicality and systematicity.

This concept of the economic burden of disease broadly encompasses the direct, indirect, and intangible economic impacts that diseases exert on individuals, households, and society as a whole. Households face a particular severe issue when bearing the substantial costs associated with disease treatment (3). This not only increases their economic burden but also exposes them to a higher risk of poverty (4). Focusing on health challenges that are under-documented or overlooked is at the core of the value of GBD research (5).

Globally, opportunities for health insurance among migrant populations are often constrained, leading to an insufficient focus on these groups in current GBD research (6). In China, the mass migration of rural workers to urban areas positions them on the periphery of society, yet they serve as the primary economic support for their rural households (7). These workers frequently need to provide regular financial assistance to their families (8). Although remittances can sustain the livelihoods of their families to some extent, the healthcare costs for family members severely disrupt the living and employment conditions of rural migrant workers (9). Consequently, household medical expenses often become the “last straw” that breaks the camel’s back for rural migrant workers, exposing them to the severe threat of poverty (10).

When it comes to measuring the economic burden of disease, various studies have focused on standardizing cost assessments (11, 12). However, it’s important to note that “cost” and “burden” carry different connotations. Objective measures of disease treatment costs have limitations in defining the subjective concept of “burden” (13). The same cost may be a burden for low-income individuals but not a substantial issue for high-income individuals. As a result, each person’s perception of disease and economic burden may differ (14).

For China’s urban and rural migrant workers, spatial mobility often leads to changes in family strategies and can have negative repercussions (15). Due to their exclusion from the system and the unequal access to medical services in China, the cost of treating illnesses among family members becomes an overwhelming financial burden for migrant workers. Even if their families have basic medical insurance, it still adds to the financial strain experienced by migrant workers (16).

Our research revealed that the requirement for medical treatment for family members adds to the financial burden and creates diverse pressures for migrant workers. It can be said that migrant workers experience a unique phenomenon of “financial toxicity.” Women, in particular, may be disproportionately affected by these challenges (17). Compared to urban residents, migrant women face a more pronounced and complex “work-family” double burden (18). However, this aspect remains relatively understudied in the field of immigration research.

This article makes a dual contribution. Firstly, it examines the relationship between the economic burden arising from family member illness (EBFI) and poverty among young migrant workers. Secondly, it recognizes the potential influence of gender as a key factor shaping the economic burden of disease. In March 2024, the World Health Organization published a significant report titled “A fair share of health and care: Gender and the undervaluation of health and care work.” This report highlights that inadequate investment in health systems creates a detrimental cycle of unpaid health and care work, diminishes women’s involvement in the paid labor market, undermines their economic empowerment, and hampers gender equality (19). Aligned with this recommendation, our study delves into gender disparities in the impact of EBFI on poverty. By doing so, we offer a fresh lens through which to examine the phenomenon of “financial toxicity” experienced by migrant groups, while also shedding light on the social gender role perceptions prevalent among young migrant workers.

## 2 Literature review and research hypotheses

### 2.1 Economic burden of disease and poverty of migrant workers

The financial burden of disease encompasses the challenges individuals face in paying for medical bills and accessing necessary healthcare services (20). It is widely recognized that illness represents a significant factor driving vulnerable families into poverty, with medical debt being the leading cause of household bankruptcy (21). In China, nearly 50% of the impoverished population can be attributed to individuals trapped in poverty due to medical expenses (National Administration for Rural Revitalization (22)). The escalating costs of medical care have a profound impact on the well-being of families.

Particularly, in rural households where the head of the household is often older adult, there is at least one family member with poor health, or some adults lack health insurance, the likelihood of being impoverished by illness is heightened. Furthermore, rural China exhibits low medical insurance coverage rates and reimbursement ratios (23). When families encounter difficulties in covering the expenses of disease treatment, migrant workers shoulder the burden, thereby facing an immense financial strain.

Family systems theory posits that the family functions as a complex social system wherein its members are interconnected, interdependent, and exert influence on each other’s behaviors (24). According to this theory, the relationship between hardship, illness, and family well-being is not unidirectional. Instead, these connections operate within a mutually reinforcing system in which stress and disruptions in one area simultaneously impact other aspects (25). In other words, changes occurring in a single family member, be it a parent, child, or sibling, can have ripple effects throughout the entire system, leading to subsequent changes in other family members (26).

Within China’s social discourse system, social isolation and poverty are commonly seen as the norm and fundamental aspects of migrant workers’ lives (27). Migrants who have family members requiring costly treatment face the unjustness of the medical insurance system and the absence of social security, which places the financial burden of the disease squarely on their shoulders. These individuals may encounter the “double adaptation burden” of fulfilling caregiving responsibilities for their family members while also navigating the challenges associated with their immigration status (18). The interplay between these two roles exacerbates their overall burden and adds to the stress they experience in their daily lives (28).

In terms of research methods, numerous current studies employ objective medical expenditures as independent variables to examine the influence of out-of-pocket medical costs or catastrophic medical expenses on income, employment, and family well-being (29, 30). While assessing the objective cost of disease is valuable and necessary, it is important to acknowledge that the affordability of medical expenses varies among families due to differing economic circumstances.

Objective medical costs alone cannot capture the subjective perceptions individuals hold regarding the financial burden of their family members’ disease treatment expenses (31). Exploring self-assessed burden, whether financial or caregiving, is a valuable measure that can yield varying results depending on the specific disease situation, future prospects, or strategic responses (32), thereby providing additional insights for research purposes (33, 34). This holds true for the economic burden of disease as well. Consequently, our study addresses this gap, offering a valuable direction for future investigations into a more comprehensive understanding of the economic burden of disease.

### 2.2 Gender inequality in economic burden of disease

The process of urbanization and changes in socioeconomic structure have had an impact on gender inequality, albeit to a certain extent (35). China has witnessed remarkable economic development over the past four decades, particularly following its reform and opening up. This growth has been characterized by increased female labor force participation, rapid urbanization, and significant rural-to-urban migration (36). However, there has been a reduction in state support for child and elder care, leading to a notable transfer of caregiving responsibilities to families (37). Although the proportion of women engaged in paid work in China is relatively higher compared to other parts of the world, they continue to bear a disproportionate burden of unpaid work (68). Women face mounting pressure to juggle the dual roles of breadwinner and family caregiver.

Migration plays a significant role in reshaping the division of gender roles within society (38). In recent decades, the proportion of female migrant workers has steadily increased (39). Throughout the migration process, traditional gender roles undergo redefinition, as some women experience enhanced economic independence. However, this newfound independence often comes at a cost (40).

Migrant women, in particular, face a more pronounced and intricate double burden compared to their urban counterparts (18). Female migrant workers not only face the substantial economic costs associated with urban living but also shoulder the financial support and caregiving duties for their families. Consequently, they face a heightened risk of poverty. It is crucial to recognize that migration and its consequences have distinct impacts on men and women (41). Our study aims to bridge this gap and provide a comprehensive understanding of the issue.

### 2.3 Analysis framework

In summary, previous research has made significant progress in examining the economic burden of disease and poverty among migrant workers, but there are still several outstanding areas that require attention. Firstly, migrant workers exhibit a higher vulnerability in their livelihoods. Hence, they face a unique risk of “economic toxicity” compared to non-migrants. Although existing studies have analyzed the poverty of migrant workers from multiple dimensions, there is a lack of consideration from a systematic family perspective. Secondly, there has been limited exploration of gender differences among migrant workers in existing research. What are the differences in the economic burden of disease and poverty between male and female young migrant workers? Are there gender differences in the impact of the economic burden of disease on poverty? Our study innovatively addresses these gaps.

**Figure 1:**
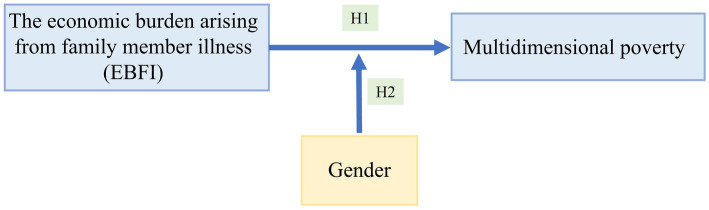
Analytical framework.

Therefore, this study empirically analyzes the influence of the economic burden arising from family member illness on the multidimensional poverty of young migrant workers, while also examining the gender differences in this impact. Drawing on existing literature, we propose the following two research hypotheses. Please refer to Figure 1 for the analytical framework.

*H1*: The economic burden arising from the illness of family members contributes to an increased risk of poverty among young migrant workers.

*H2*: A significant gender disparity exists in the impact of the economic burden arising from family member illness on the poverty levels of young migrant workers. Specifically, this burden has a more pronounced positive effect on the poverty risk of female young migrant workers compared to their male counterparts.

## 3 Research design

### 3.1 Data

The data used in this study comes from the China Migrant Dynamic Survey (CMDS) conducted in 2017. The CMDS is an annual large-scale sampling survey conducted by the National Health Commission of China on domestic migrants, covering 31 provinces, autonomous regions, municipalities directly under the Central Government, and the Xinjiang Production and Construction Corps in mainland China. Through stratified sampling and multi-stage probability proportional to size (PPS) methods, the CMDS collected data on the floating population aged over 15 who had lived in the receiving city for more than 1 month and did not have local household registration, with a total sample size of 169,989 individuals. The PPS method implies that the probability of selecting each unit in each sampling is proportional to the size of the unit. Therefore, its characteristic is that the probability of most of the content of the whole being sampled can improve the representativeness of the sample. The 2017 survey was stratified by province (autonomous region, municipality directly under the Central Government), city or district, township (or street), and village (or residents’ committee). Within each stratum, sampling was conducted according to the PPS method, and finally, the sample floating population was mapped (42). Auxiliary information about population size was used at each level and at each stage to reduce sampling errors (43).

The CMDS survey is the largest and most representative database of the floating population in China. The sampling methods it employs and the collected survey data are highly representative, making it suitable for conducting research related to Chinese migrant workers. Referring to the “Migrant Workers Monitoring Survey Report” published by the National Bureau of Statistics of China,^1^ this study defined young migrant workers as domestic migrants aged 18–35 with rural household registration and engaged in non-agricultural work in urban areas. Consequently, unemployed and underemployed samples were excluded. Given the extensive size of the CMDS database, samples with missing information were removed. As a result, a total of 36,931 valid samples were obtained for analysis.

### 3.2 Variables

#### 3.2.1 Independent variable: the economic burden arising from family member illness (EBFI)

The main objective of this article is to investigate the economic burden arising from family member illness (EBFI). Taking into account the unique circumstances of migration and the data at hand, our focus centers on the perceptions of individual migrant workers regarding the financial challenges faced by their family members due to illnesses. In the CMDS survey, respondents were posed with the following question: “Do you have any family members who are currently sick and facing financial constraints in accessing medical treatment?” For analysis purposes, a value of 1 was assigned to respondents who answered “yes,” while a value of 0 was given to those who responded with “no.”

#### 3.2.2 Dependent variable: multidimensional poverty

Drawing upon Amartya Sen’s capability theory, poverty is conceptualized as the lack of essential capabilities needed for a decent life (44). Currently, numerous studies rely on the Multidimensional Poverty Index (MPI) developed by the United Nations Development Program to establish a comprehensive indicator system for measuring multidimensional poverty. Initially, the MPI was primarily employed to gage poverty among rural households. However, its direct application to measure the multidimensional poverty of migrant workers may introduce potential biases.

Existing research has shown that poverty measurement based on households underestimates the overall level of social poverty, as it fails to capture the distribution of resources and inequality within the household (45). In 2030, the Sustainable Development Agenda advocated for examining poverty at the individual level, that is, “leaving no one behind” (46). Therefore, our study selected indicators at the individual level of migrant workers to measure their poverty and adjusted the measurement methods accordingly.

The initial MPI index only included three dimensions: health, education, and standard of living. Sen proposed the income-capability supplement method. This is a non-radical and practical approach that uses income as a traditional measurement standard and uses capability consideration as a supplement. This provides a more applicable measurement scheme for poverty analysis and also provides a more interpretive measurement standard for policy analysis (47).

Therefore, based on Sen’s capability theory and taking into account previous research and the specific circumstances of Chinese migrant workers, we extended the Multidimensional Poverty Index (MPI) and devised a comprehensive framework from five dimensions to measure the multidimensional poverty of young migrant workers. We employed the equal weighting method for setting weights. Detailed explanations of dimensions and indicators can be found in Table 1.

**Table 1:** Multidimensional poverty index system and threshold of young migrant workers.

Dimension	Index	Threshold value	Weight
Income	Wage	Annual wage income is less than 50% of the disposable income of urban residents in 2017 = 1	0.2
Education	Education	Did not complete junior high school =1	0.2
Employment	Working stability	No labor contract has been signed = 1	0.1
	Labor time	Working more than 44 h a week = 1	0.1
Health	Seek medical attention for illness	Illness (injury) or physical discomfort (medical treatment due to illness) in the last year = 1	0.05
	Self-rated health	Self-rated health status as “unhealthy” = 1	0.05
	Medical insurance	No health insurance whatsoever = 1	0.05
	Health file	No resident health records were established =1	0.05
Livelihood	Employment difficulty	It’s hard to find steady work = 1	0.05
	Financial difficulty	Low income = 1	0.05
	Housing difficulties	Cannot afford to buy a house = 1	0.05
	Social exclusion	Experienced being ostracized by the locals = 1	0.05

#### 3.2.3 Control variables

Drawing upon relevant literature, this article considers several controlled variables encompassing the demographic characteristics, occupational characteristics, and regional factors of young migrant workers. Specifically, the control variables include: gender, age, education, marital status, industry, occupation, working years, income, migration frequency, migration distance, housing prices, rural homestead ownership, and region. The explanations and descriptive statistics for all variables are presented in Table 2.

**Table 2:** Variable description.

Variables	Explanation	Mean	Std.	Min	Max
EBFI	Have the economic burden arising from family member illness = 1, Otherwise = 0	0.1186	0.3234	0	1
Multidimensional poverty status	Multidimensional poverty = 1, Otherwise = 0	0.3093	0.4622	0	1
Multidimensional poverty level	Multidimensional Poverty Index	0.3749	0.0876	0.3	0.9
Age	Actual age (years)	27.44	4.58	18	35
Age^2^	The square of actual age	773.93	247.16	324	1,225
Male	Male = 1, Female = 0	0.5245	0.4994	0	1
Married	Married = 1, Otherwise = 0	0.5998	0.4899	0	1
Education	Years of education	11.17	2.91	0	19
Industry	The industry of the respondents. Construction = 0; Manufacturing = 1; Service = 2; Culture, Education, Sports, Entertainment and Social Organizations = 3; Other = 4	1.61	0.89	0	4
Occupation	Occupation of the respondent. Manual workers and others = 1; Commercial service personnel = 2; Office staff = 3; Unit leaders and professional technicians = 4.	1.9205	0.9909	1	4
Working years	Actual working years	2.9152	3.1734	0	22
Income	Logarithm of actual monthly wage income	8.1152	0.7182	0	11.15
Migration frequency	Number of migrations	1.9153	1.2374	1	9
Migration distance	Migration distance. Intra-provincial migration = 0; Inter-provincial migration = 1	0.5108	0.4999	0	1
Housing price	House prices in the cities where the respondents live (logarithm)	9.3948	0.7436	7.47	10.94
Rural homestead ownership	Have a homestead in the countryside = 1, Otherwise = 0	0.6802	0.4664	0	1
Region	Eastern = 4; Northeastern = 3; Central = 2; Western = 1	2.7830	1.3192	1	4

### 3.3 Method

#### 3.3.1 A-F method

Alkire and Foster were pioneers in applying Amartya Sen’s capability approach to global multidimensional poverty measurement through the counting method, known as the “A-F” method. This method establishes poverty by setting two crucial values. Firstly, if an individual falls below the threshold in a specific dimension, they are considered deprived in that dimension. Secondly, the deprivation threshold (*k*) for multidimensional poverty is determined based on the total number of dimensions in which an individual is deprived. Currently, existing studies commonly employ a threshold of *k* ≥ 0.3 or *k* ≥ 1/3 to ascertain whether the subjects under investigation are experiencing multidimensional poverty, using this as the foundation for subsequent empirical analysis. In accordance with this criterion, this study defines young migrant workers with *k* ≥ 0.3 as being in a state of multidimensional poverty. By considering both the deprivation status in individual dimensions and the overall extent of deprivation, the “A-F” method employs a dual threshold approach to measure multidimensional poverty at the individual level.

Once the deprivation in each dimension has been identified, it becomes crucial to employ the counting method to aggregate the dimensions and obtain a comprehensive measurement of multidimensional poverty. Alkire and Foster introduced a methodology that comprises two components for the multidimensional poverty index: poverty incidence (H) and average deprivation score (A). The formula for calculating these components is as follows: M = HA.

M is the multidimensional poverty Index. The poverty incidence (H) is computed by dividing the number of deprived samples (*q*) by the total sample size (*N*). This calculation provides a measure of the proportion of the population living in poverty. The average deprivation score (A), also referred to as the poverty intensity index, is determined by dividing the average number of dimensions in which all poor individuals are deprived by the total number of dimensions (*z*). Furthermore, the multidimensional poverty index can calculate the contribution rate (*β*) of each dimension according to different characteristics (48).

#### 3.3.2 Model setting

The dependent variable in this study is multidimensional poverty, which we refer to in terms of its status and severity. The status of multidimensional poverty is represented as a binary variable; thus, we employ a binary logistic regression model. For samples in a state of multidimensional poverty, we calculate their level of multidimensional poverty, known as the MPI index. According to the A-F method, the MPI index is calculated based on the subset of samples where the multidimensional poverty status is 1 (*k* ≥ 0.3); samples with *k*<0.3 are not considered impoverished. Consequently, the MPI index is constructed using truncated data with a left truncation point of 0, for which we employ the Tobit model.

## 4 Results

### 4.1 The economic burden arising from family member illness among young migrant workers

**Figure 2:**
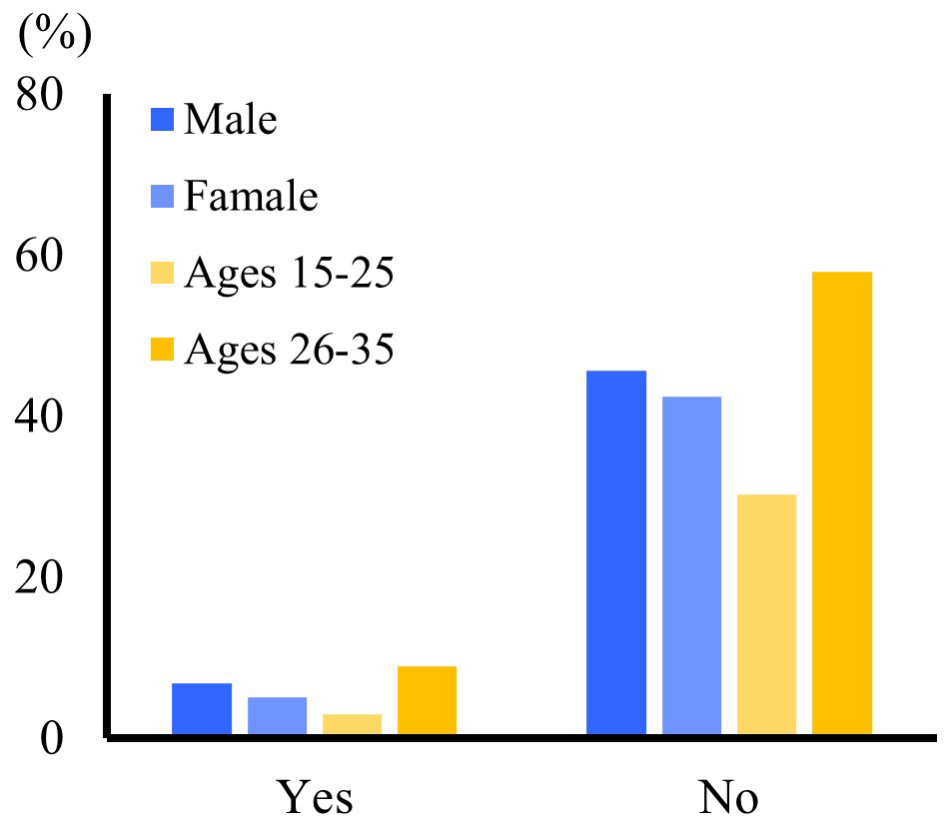
Gender and age comparison of disease economic burden of young migrant workers.

The gender and age comparisons of the economic burden arising from family member illness (EBFI) among young migrant workers are presented in Table 3 and Figure 2. The results of descriptive statistical analysis and chi-square tests showed the significance of gender and age differences in EBFI among young migrant workers.

**Table 3:** Gender and age comparison of disease economic burden of young migrant workers.

	Yes	No	Total	Chi-square test
Male	2,507 (6.79%)	16,865 (45.67%)	19,372 (52.45%)	45.3442***
Female	1874 (5.07%)	15,685 (42.47%)	17,559 (47.55%)	
Total	4,381 (11.86%)	32,550 (88.14%)	36,931	
Ages 18–25	1,079 (2.92%)	11,166 (30.23%)	12,245 (33.16%)	163.0849***
Ages 26–35	3,302 (8.94%)	21,384 (57.90%)	24,686 (66.84%)	
Total	4,381 (11.86%)	32,550 (88.14%)	36,931	

In terms of gender, EBFI is significantly higher among men (6.79%) compared to women (5.07%) within the young migrant workers. Regarding age, there is a significant discrepancy in EBFI across different age groups. Migrant workers aged 26–35 experience a considerably higher EBFI (8.94%) compared to those aged 18–25 (2.92%).

### 4.2 Multidimensional poverty measurement results

#### 4.2.1 Overall multidimensional poverty situation

**Figure 3:**
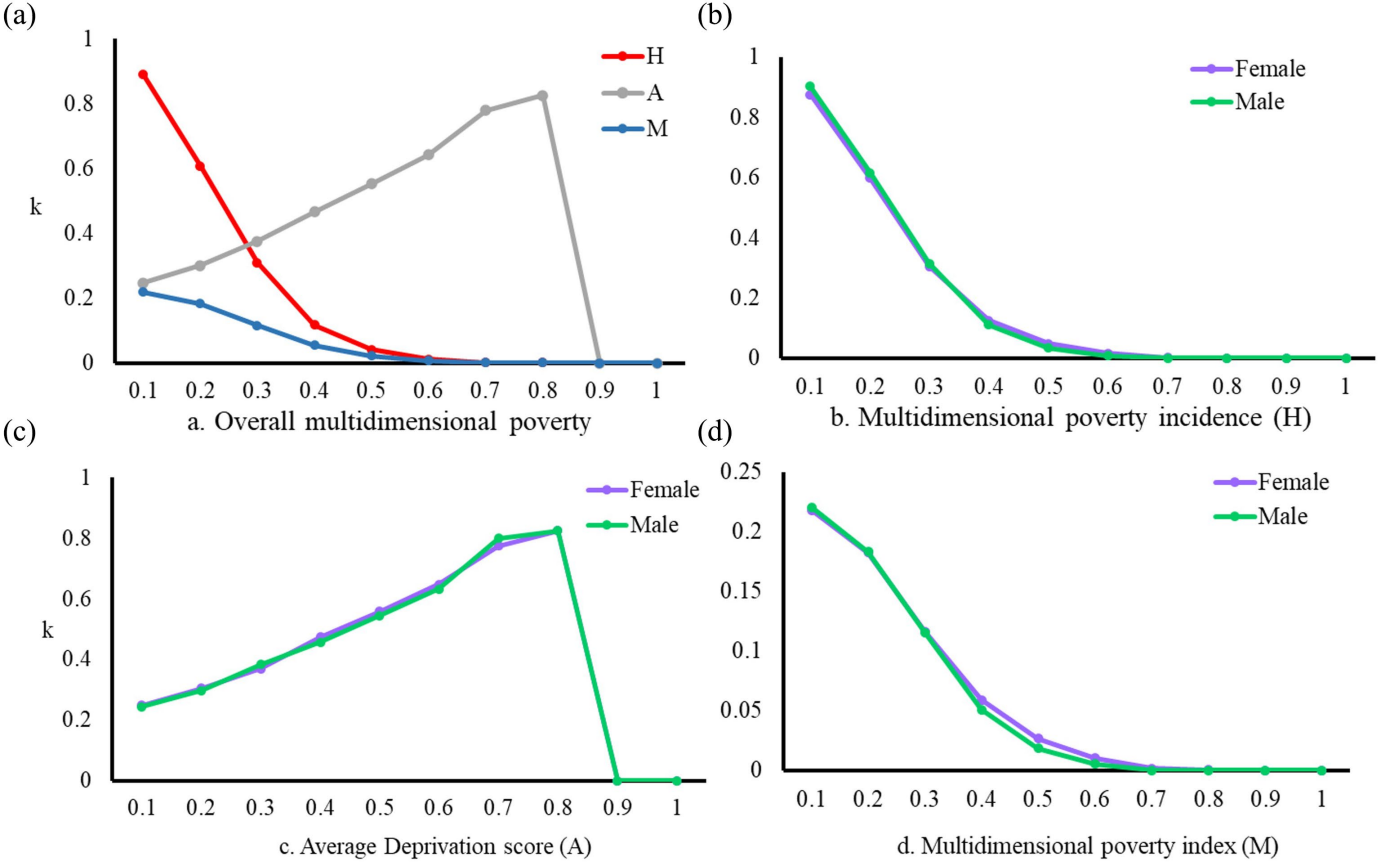
Multidimensional poverty measurement results of young migrant workers. **(A)** Overall multidimensional poverty. **(B)** Multidimensional poverty incidence (H). **(C)** Average Deprivation score (A). **(D)** Multidimensional poverty index (M).

Table 4 and Figure 3A present the findings of the multidimensional poverty measurement for young migrant workers. As the deprivation threshold (k) increases, there is a gradual decrease in the multidimensional poverty index (M) and multidimensional poverty incidence (H) among young migrant workers. Simultaneously, the average deprivation score (A) shows a gradual increase. The average deprivation score reflects the proportion of the number of deprived dimensions to the total number of dimensions. This indicates that as the k-value becomes larger, the proportion of young migrant workers experiencing multidimensional poverty diminishes, a higher number of dimensions in which them face deprivation. Consequently, the A-value rises, and the M-value tends to decrease.

**Table 4:** Multidimensional poverty measurement results of young migrant workers.

k	H	A	M
0.1	0.8897	0.2463	0.2191
0.2	0.6078	0.3006	0.1827
0.3	0.3093	0.3749	0.1160
0.4	0.1177	0.4655	0.0548
0.5	0.0405	0.5526	0.0224
0.6	0.0118	0.6422	0.0076
0.7	0.0011	0.7786	0.0009
0.8	0.0004	0.8250	0.0004
0.9	0	1	0
1	0	1	0

Currently, existing studies commonly adopt a threshold of *k* ≥ 0.3 or *k* ≥ 1/3 to determine whether the research subject is experiencing multidimensional poverty. This threshold serves as the basis for subsequent empirical analyses. According to this criterion, young migrant workers with *k* ≥ 0.3 in the sample are defined as multidimensional poverty. As a result, the statistical findings indicate that when *k* = 0.3, the H-value among young migrant workers is 30.93%, and the A-value is 37.49%. This suggests that approximately 30% of young migrant workers experience deprivation in at least four indicators. Furthermore, the M-value is calculated to be 0.1160. For cases where *k* > 0.8, the A-value is 1, and the H-value is 0. These values imply that no young migrant workers fall into the category of extreme multidimensional poverty.

Figures 3B–D illustrates the gender disparities in overall multidimensional poverty experienced by young migrant workers. From the perspective of poverty incidence (H), there is no significant difference in the levels of multidimensional poverty between male and female young migrant workers. However, upon closer examination, it is observed that as the deprivation threshold (*k*) increases within the range of 0.4 ≤ *k* ≤ 0.7, the multidimensional poverty index (M) for female young migrant workers is markedly higher than that for males. Considering the threshold employed in this study is *k* ≥ 0.3, it can be concluded that the risk of multidimensional poverty among female young migrant workers in China is higher than that among males.

#### 4.2.2 Comparison of different dimensions

**Figure 4:**
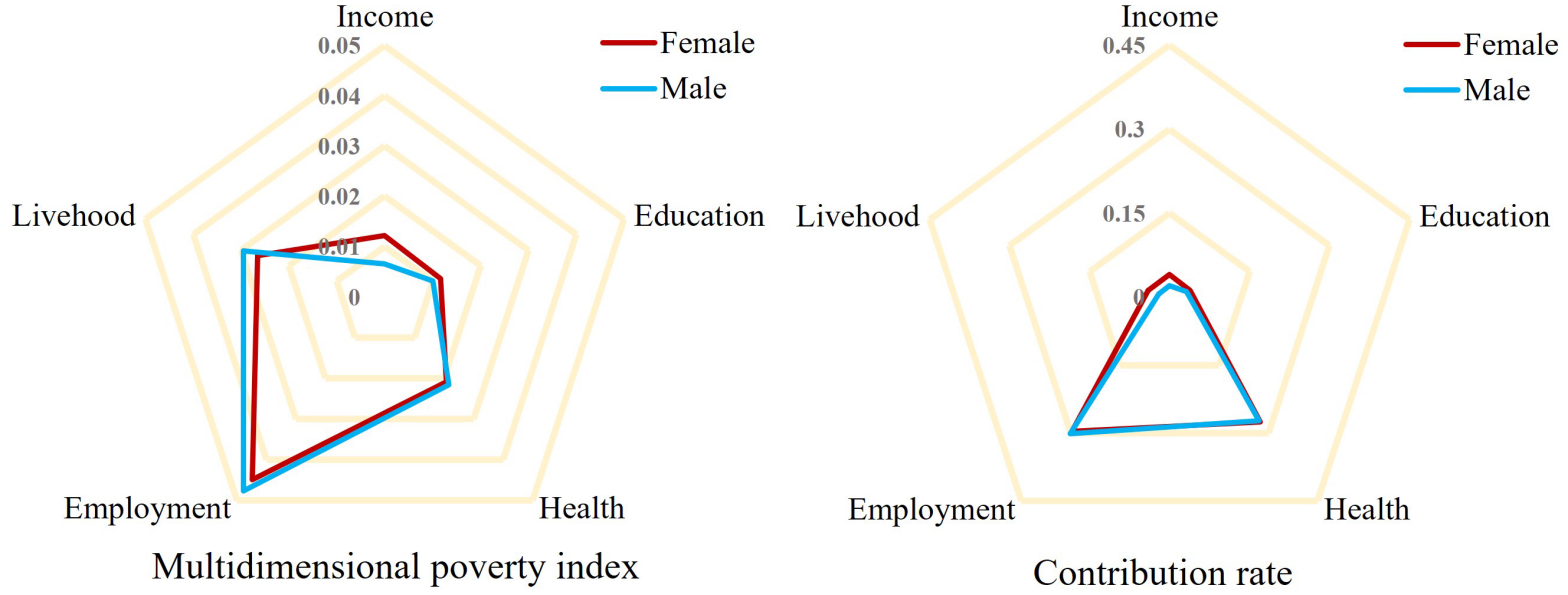
Multidimensional poverty by gender and dimensions.

Table 5 presents a comparative analysis of gender differences across various dimensions to explore the level of deprivation experienced by male and female young migrant workers. By utilizing the poverty index for each dimension, we can further determine the contribution rate (*β*) of each dimension to overall multidimensional poverty. In terms of the poverty index, male young migrant workers exhibit higher indices in the dimensions of employment, livelihood, and health compared to their female counterparts, whereas female young migrant workers display significantly higher indices in the dimensions of income and education. Regarding the contribution rate, the poverty contribution rates in the dimensions of income and living conditions are higher among females. Figure 4 visually depicts these results in a more intuitive manner.

**Table 5:** Multidimensional poverty by gender and dimensions.

	M		*β*	
Dimension	Female	Male	Female	Male
Income	0.0122	0.0065	0.0402	0.0204
Education	0.0117	0.0101	0.0384	0.0318
Health	0.0209	0.0216	0.2752	0.2727
Employment	0.0449	0.0477	0.2961	0.3011
Livelihood	0.0266	0.0296	0.0402	0.0204

### 4.3 Impact of economic burden of disease on multidimensional poverty

#### 4.3.1 Basic regression results

Following the measurement of the multidimensional poverty among young migrant workers, we proceeded to investigate the influence of the economic burden arising from family member illness (EBFI) on their multidimensional poverty. The findings are presented in Table 6. Model 1 represents a binary logit model with the multidimensional poverty status as the dependent variable. To ensure the reliability of the results, Model 2 adopts a Tobit model with the multidimensional poverty index as the dependent variable. This two-model approach enhances the robustness of our analysis and provides a comprehensive understanding of the relationship between EBFI and multidimensional poverty among young migrant workers.

**Table 6:** The impact of EBFI on multidimensional poverty.

	Model 1		Model 2	
Variables	Odds ratio	SE	Coef.	SE
EBFI	1.574***	0.056	0.006**	0.002
Age	0.957	0.028	−0.001	0.002
Age^2^	1.001	0.001	0.0000	0.000
Male	0.683***	0.031	−0.005***	0.002
Married	1.438***	0.046	0.000	0.002
Education	0.568***	0.008	−0.030***	0.001
Industry	1.030	0.015	0.005***	0.001
Occupation	1.011	0.014	0.001	0.001
Working years	1.004	0.044	0.000	0.000
Income	0.474***	0.014	−0.019***	0.001
Migration frequency	1.050***	0.008	0.000	0.000
Migration distance	1.042**	0.013	−0.001	0.001
Housing price	1.250***	0.033	0.005**	0.002
Rural homestead ownership	0.754***	0.018	−0.006***	0.001
Region	1.041	0.030	−0.004*	0.002
Constant	15,803.513***	7157.669	0.678***	0.026
Observations	36,931		11,423	
LR Chi^2^	4852.34		2646.04	
pseudo-R	0.1062		0.1140	

*p < 0.05, **p < 0.01, ***p < 0.001; Standard error in parentheses.

The findings from Model 1 demonstrate that the EBFI has a significant impact on the incidence of experiencing multidimensional poverty among young migrant workers. Specifically, the odds of multidimensional poverty are 1.574 times higher for migrant workers with EBFI compared to those without EBFI. Furthermore, the results from Model 2 reveal that EBFI plays a crucial role in exacerbating the depth of multidimensional poverty among young migrant workers. These results provide robust evidence, reinforcing the validity and consistency of the findings observed in Model 1.

Regarding the control variables, it is observed that female and married young migrant workers are more susceptible to experiencing multidimensional poverty compared to their male and unmarried counterparts. Furthermore, young migrant workers with lower levels of education, lower income, higher frequency of mobility, longer migration distances, and higher urban housing prices face a significantly elevated risk of falling into multidimensional poverty. Additionally, the ownership of contracted land in rural areas is also identified as a variable that significantly affects the multidimensional poverty status of young migrant workers.

#### 4.3.2 Robustness test: propensity score matching method

**Figure 5:**
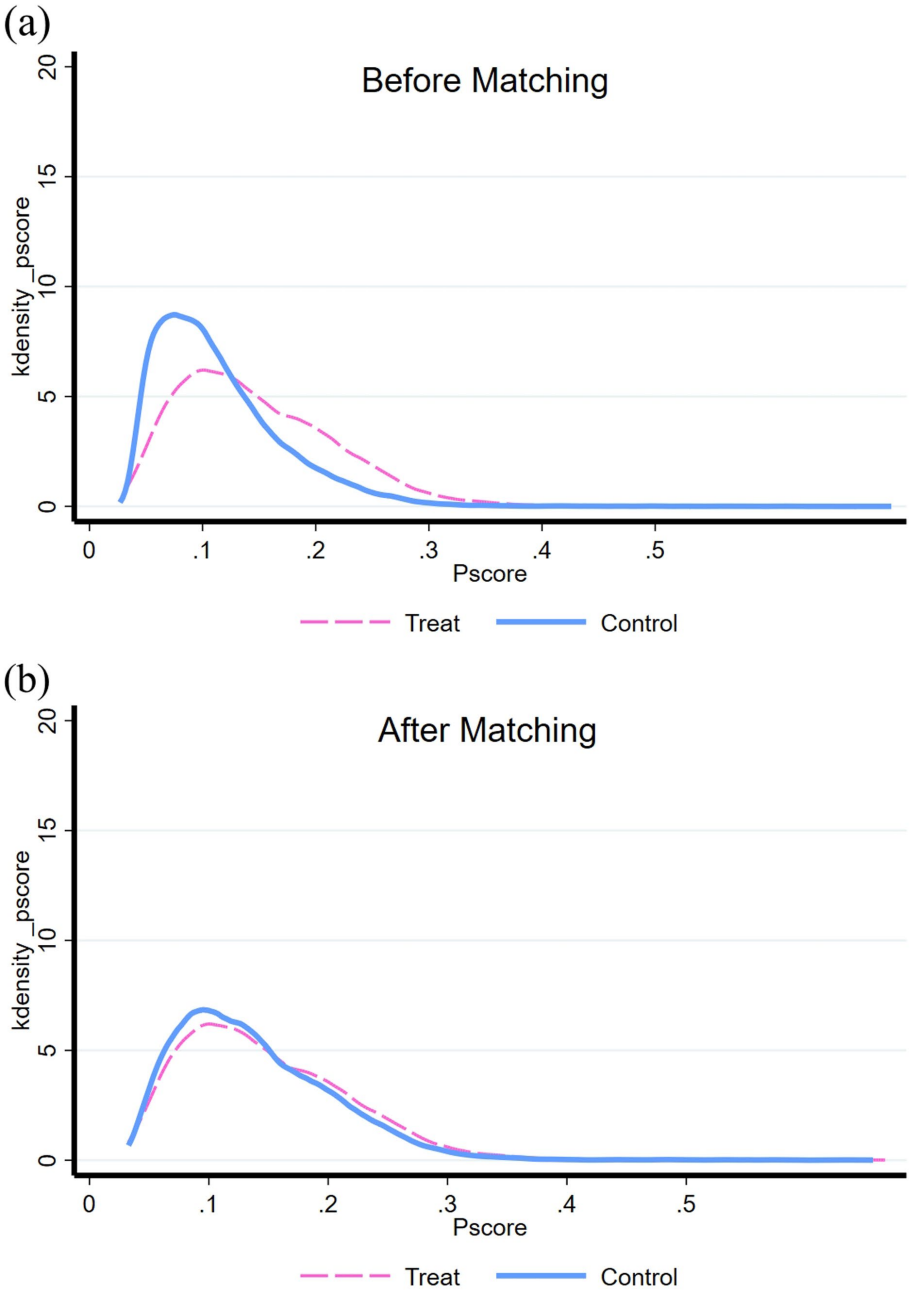
Propensity score density distribution before and after nearest neighbor 1:1 matching. **(A)** Before matching. **(B)** After matching.

It is important to note that EBFI is not distributed randomly but influenced by various factors. To mitigate the potential influence of sample selection bias on statistical outcomes, we employ the propensity score matching method for robustness testing. This approach helps address any potential biases and strengthens the reliability of our findings. The balance test serves as a crucial prerequisite for employing the propensity score matching method. In this study, we utilize the widely adopted nearest neighbor 1:1 matching method. Figure 5 depicts a propensity score density map.

Prior to matching, there existed a substantial disparity in the propensity score density distribution between young migrant workers with EBFI (experimental group) and those without EBFI (control group), indicating the presence of a selective mechanism between the two groups. However, following the matching process, the propensity score density distribution diagrams of the experimental and control groups became highly similar. This indicates that the propensity values pertaining to EBFI were comparable between the two groups, resulting in improved balance and more favorable propensity score matching outcomes.

Upon assessing the data balance test results after employing the nearest neighbor 1:1 matching, it is evident that most of the confounding variables exhibited significant differences between the two groups before matching (Figure 5A). However, after matching (Figure 5B), nearly all variables no longer exhibited significant differences between the two sample groups (with *p*-values greater than 0.05). This signifies that the propensity score matching achieved sample balance by effectively reducing the systematic differences between the experimental and control groups.

The model results after applying propensity score matching is presented in Table 7. The findings indicate that using the nearest neighbor 1:1 matching method, the Average Treatment Effect on the Treated (ATT) value is estimated to be 0.1035, with a *t*-value of 10.73. This result is statistically significant at the 1% level. These findings affirm that, after accounting for sample selection bias, EBFI has a positive impact on the multidimensional poverty risk among young migrant workers. This outcome provides empirical support for hypothesis H1, confirming the association between EBFI and the heightened risk of multidimensional poverty among young migrant workers.

**Table 7:** The result of propensity score matching (*n* = 36,931).

	Dependent variable: multidimensional poverty					
Independent variable		Treated	Controls	ATT	S.E.	T
EBFI	Unmatched	0.4419	0.2915	0.1504	0.0074	20.34
	Matched	0.2915	0.3942	0.1035	0.0096	10.73

### 4.4 Moderating effect of gender

#### 4.4.1 Basic regression results

We additionally examine the moderating effect of gender. Model 3, Model 4, and Model 5 present the results of logistic regression analysis, incorporating the interaction term of EBFI and gender (Table 8). Considering that youth represent a transitional phase within a broader age range, the sample was divided based on age. Specifically, Model 3 encompasses the entire sample of young migrant workers, Model 4 focuses on young migrant workers aged 18–25, and Model 5 concentrates on young migrant workers aged 26–35. These models allow us to delve deeper into the moderating influence of gender on the relationship between EBFI and multidimensional poverty among young migrant workers across different age groups.

**Table 8:** Logistic regression results of cross-multiplication terms.

	Model 3		Model 4		Model 5	
Variables	Odds ratio	SE	Odds ratio	SE	Odds ratio	SE
EBFI	1.732***	0.093	1.626***	0.159	1.813***	−0.118
EBFI*Male	0.845*	0.060	0.993	0.141	0.798**	0.067
Age	0.956	0.028	0.948	0.156	1.146	0.143
Age^2^	1.001	0.001	1.001	0.004	0.998	0.002
Male	1.006	0.068	1.168	0.158	0.930	0.073
Married	1.439***	0.046	1.471***	0.087	1.423***	0.057
Education	0.568***	0.008	0.604***	0.017	0.549***	0.009
Industry	1.029	0.015	1.056	0.032	1.013	0.018
Occupation	1.011	0.014	1.005	0.028	1.001	0.017
Working years	1.025***	0.004	1.054***	0.013	1.019***	0.004
Income	0.474***	0.014	0.245***	0.015	0.602***	0.019
Migration frequency	1.050***	0.008	1.075***	0.018	1.043***	0.009
Migration distance	1.042**	0.013	1.040+	0.024	1.050**	0.016
Housing price	0.754***	0.018	0.792***	0.034	0.740***	0.021
Rural homestead ownership	1.250***	0.033	1.192***	0.057	1.261***	0.04
Region	1.041	0.030	1.133*	0.059	0.999	0.035
Constant	15,795.432***	7,154.63	1152506.831***	2071952.1	217.419**	416.172
Observations	36,931		12,245		24,686	
LR Chi^2^	4857.84		1732.82		3312.13	
pseudo-R	0.1063		0.1177		0.1071	

*p < 0.05, **p < 0.01, ***p < 0.001; Standard error in parentheses.

Analyzing the results of Model 3, it is evident that the estimated coefficient of the interaction term between the economic burden arising from family member illness (EBFI) and gender is negative. These statistical findings demonstrate significance at the 5% level, indicating that male young migrant workers face a significantly lower risk of experiencing multidimensional poverty as a result of EBFI compared to their female counterparts. Stated differently, women are more susceptible to the impact of EBFI within their families, leading to a higher likelihood of falling into multidimensional poverty. Consequently, hypothesis H2 is confirmed, providing empirical support for the assertion that gender plays a moderating role in the relationship between EBFI and multidimensional poverty among young migrant workers.

The results of Model 4 reveal that the inclusion of the interaction term does not yield statistical significance among the 18–25 age group. This suggests that the relationship between the EBFI and multidimensional poverty among young migrant workers aged 18–25 is not significantly influenced by gender. However, in Model 5, it is observed that among young migrant workers aged 26–35, the probability of men experiencing multidimensional poverty as a result of EBFI is significantly lower compared to women by 20.2%. These findings indicate that gender plays a significant moderating role only within the sample of young migrant workers aged 26–35.

Overall, the results from the three models demonstrate that the moderating effect of gender is significant solely among the subset of young migrant workers aged 26–35. Age appears to be an important factor in understanding the interplay between gender, EBFI, and multidimensional poverty among young migrant workers.

#### 4.4.2 Robustness check: reprocessing of interaction terms

In accordance with Ai and Norton (49), it is essential to note that the interpretation of interaction terms in nonlinear regression models differs from that in linear models. In the context of logistic regression model results, merely analyzing the coefficient and t-test results of the interaction term does not provide a comprehensive understanding of the marginal effect and significance level associated with the interaction term.

**Figure 6:**
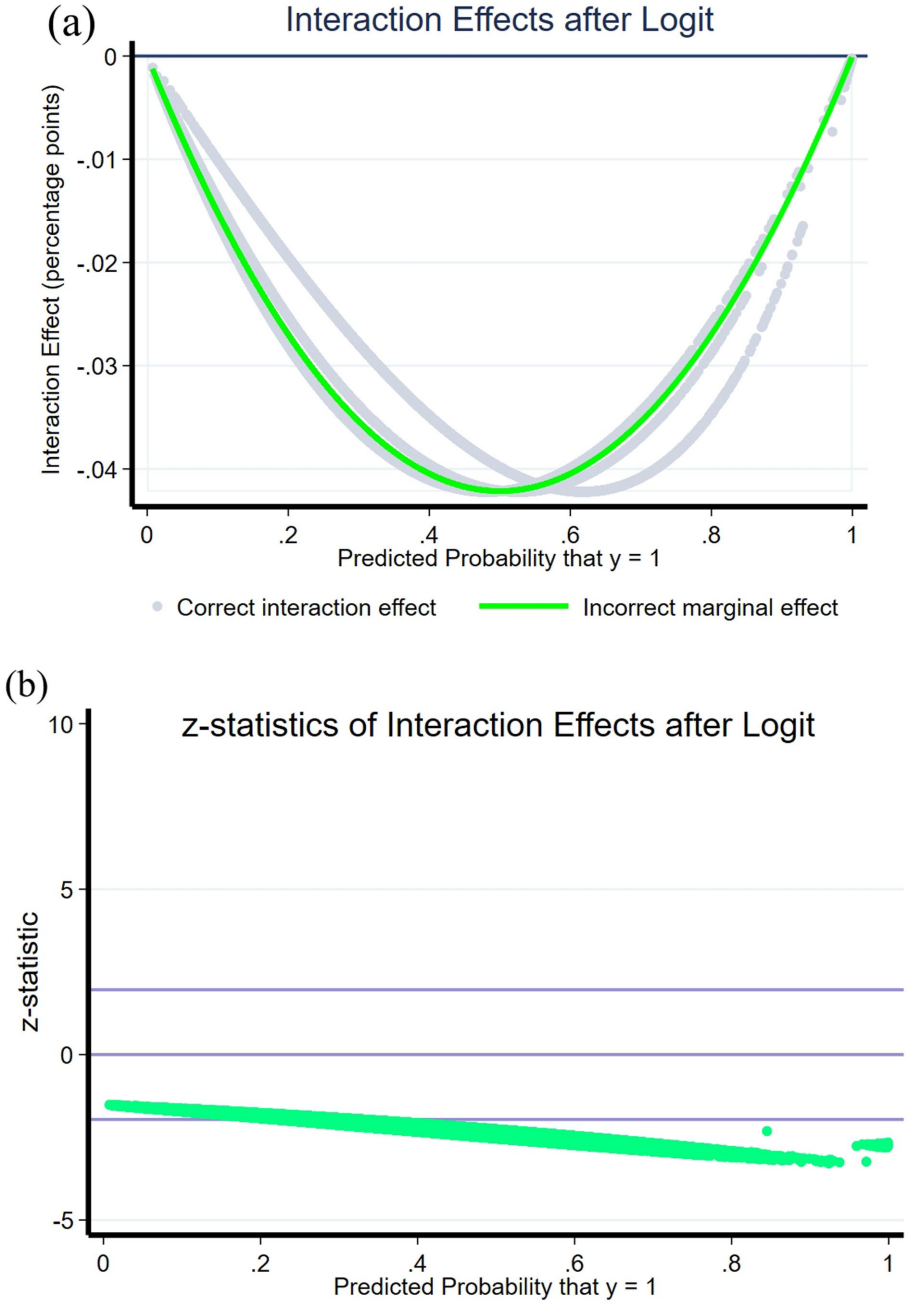
Robustness test of interaction effects. **(A)** Interaction effects after logit. **(B)** *z*-statistics of interaction effects after logit.

To assess the robustness of the moderating effect, we turn to the study conducted by Norton et al. (50) for guidance. Following their methodology, we utilize the “inteff” command in Stata to compute the marginal effect of the interaction term between EBFI*Male. Additionally, we obtain the Z statistic to perform a comprehensive robustness test. In Figure 6A, we present a comparison between the erroneous marginal effect of the interaction term derived from the linear model and the accurate marginal effect derived from the nonlinear model.

Upon utilizing Norton, E. C’s methodology, it is evident that the results of the two marginal effects exhibit a high level of consistency. Across almost all samples, the interaction effect is consistently negative, aligning with the direction of the interaction term coefficient observed in Model 3. In Figure 6B, the band-shaped area formed by the two straight lines above and below represents the Z-value interval at the significant level, indicating the passing of the statistical test. The significance of the result is determined by the majority of points falling within this interval. The findings indicate that, except for a few data points with incidence rates between 0 and 0.2, the interaction effects remain significant across most samples. This outcome aligns with our earlier model results. Our moderation results are robust.

## 5 Discussion

### 5.1 The unique “financial toxicity” of young migrant workers in China

Following the initiation of market-oriented economic reforms in 1978, China underwent a significant social transformation that resulted in a decline in the traditional practice of adult children providing filial care for their elders (51). Many rural residents have migrated to urban areas in search of employment opportunities. Consequently, geographical distance has emerged as a significant barrier for migrant workers in fulfilling their responsibilities toward caring for their parents and families.

He et al. (52) conducted a study revealing that the illness of migrant workers’ parents has a distinctive impact on their lives. Firstly, the treatment of illness significant financial strain on the migrant workers. Secondly, because of the precariousness of employment, migrant workers often lack the support and flexibility to take leave from their jobs in factories or small private enterprises to attend to their parents’ health needs. Thirdly, the cities where migrant workers are employed are quite far away from their hometown, making it challenging for them to provide adequate support and care. These challenges are further compounded for female migrant workers, who face additional hurdles in fulfilling their filial obligations due to the demands of long-distance travel (52).

Migrant workers face significant challenges when it comes to caring for their families and providing financial support. Similar to the concept of “financial toxicity” associated with cancer, the economic burdens experienced by migrant workers stem from institutional disparities and inadequate social security systems. These burdens extend beyond the objective costs of medical treatment and caregiving, affecting their work, livelihoods, and overall well-being. Our research findings concur with this perspective.

### 5.2 Poverty and gender differences among young migrant workers

Since 2000, female migrants have consistently constituted nearly 50% of the global migrant population (53). Consequently, the integration of a gender perspective into the examination of migration and health is imperative (54). Whether in international migration or in the urban–rural migration within China, females serve as both mobile workers and women, facing dual segregation in the labor market. Gender inequalities stem from productive labor (remunerated work) and unpaid labor (domestic chores and caregiving), leading to distinct roles and responsibilities for men and women, with differing health risks associated. Migration behaviors impact gender divisions. Some studies have found that female migrants bear more stress and caregiving burdens in both work and family spheres compared to males (both objectively and subjectively), affecting their quality of life and mental and physical health (55, 56).

Our research indicates that female migrant workers, despite gaining partial economic independence, do not completely challenge traditional gender norms. Based on our previous analysis, we found that female migrant workers bear a smaller economic burden due to family member’s illness (EBFI). However, their multidimensional poverty incidence is slightly higher than that of male migrant workers. They are at a higher risk of falling into poverty due to EBFI. Specifically, compared to male migrant workers, young female migrant workers are more likely to become impoverished due to illness. We propose that this outcome can be attributed to the higher poverty index of female migrant workers in the income dimension when compared to men. Consequently, EBFI imposes a more substantial blow to their income, exacerbating their vulnerability. It is important to note that these findings reflect the existing gender disparities in economic well-being among migrant workers.

In recent years, the increasing number of female migrants has drawn attention, and studies have shed light on the growing reliance on women’s remittances in the context of international migration, often referred to as the “feminization of survival” (57). Wang and Tang (58) discovered that China’s migrant worker policy has resulted in the separation of traditional rural families, disrupting the normal functioning of migrant worker families and subsequently raising the living costs for migrant women in urban society (58). Our research findings align with theirs, reinforcing these observations. Within China’s urban–rural dual system, the burden of diseases faced by young migrant workers from their families, as well as the gender disparities have not received sufficient attention from the academic community. We believe that the underlying cause of these challenges lies in the gender inequality prevalent in the labor market for migrant workers and the unequal distribution of family healthcare responsibilities. Additionally, the imbalance between urban and rural areas in the national medical insurance system further exacerbates these disparities.

In addition to gender differences, family structure factors also play a pivotal role in an individual’s economic burden and risk of poverty, interacting with gender factors. However, as married migrant women, whether as wives or daughters-in-law, their family responsibilities and burdens are still trapped within the female role. They bear a greater burden of disease caregiving than men, regardless of whether this burden is tangible and objective or perceived and subjective (59). Although family and gender factors are interwoven, this study temporarily focuses on gender factors because gender roles and responsibilities have significant differences in contemporary society, and these differences directly affect individual well-being. Additionally, gender factors have a clear directional significance in policy-making and intervention measures, which helps to address the specific challenges faced by young female migrant workers.

### 5.3 The role of social security

Social insurance plays a crucial role in mitigating the vulnerability of households to poverty, and increased investment in social security can help reduce the burden of disease (60). Extensive research and analysis have revealed that while medical insurance programs are important, they alone are insufficient to shield poor households from the economic burdens associated with healthcare expenses, particularly in rural areas (61). This disparity arises because the state’s coverage only extends to a portion of the medical costs, leaving individuals and families burdened with substantial non-medical expenses such as transportation, accommodation, and nutritional supplements (62). This situation is particularly challenging for those from impoverished backgrounds.

Shockingly, a survey found that 20.3% of migrant worker families have at least one individual lacking basic medical insurance (63). It is disheartening to observe that in Chinese society, both farmers and migrant workers, two vulnerable groups, often lack adequate support. When one party within a family faces economic hardships due to illness, the other members are susceptible to being trapped in a detrimental cycle of “burden of disease-poverty” due to insufficient risk resistance. The state’s exploitation of migrant workers effectively undermines their capacity to fulfill their responsibilities toward their rural family members. In practice, achieving fairness and justice in the healthcare sector appears challenging. The ideal of providing equal quality healthcare based on individual needs becomes unattainable when social resources are limited (64). The primary economic responsibility for healthcare inevitably falls on the family. In the realm of healthcare, the state can never replace a loving family in delivering appropriate care to patients (65).

A recent study has found that the potential burden of older adult care within households significantly affects family investment behavior, and a higher degree of social security participation in households can moderate and mitigate the negative impact of this burden (66). Effective social security policies are crucial for balancing the supply structure of the labor market while preventing the decline of social welfare levels. This is a significant strategic demand for social development in countries facing the pressures of family-based older adult care and the acceleration of population aging (67). In other countries with a similar culture of filial piety, like Singapore, various tax reduction and incentive measures have been implemented to enhance intergenerational cohesion and support for family caregiving. In certain regions of China, such as Beijing and Shanghai, sporadic policies and laws have been introduced to provide direct and indirect economic assistance to family caregivers. However, these measures primarily target urban populations, leaving the migrant workers largely overlooked by the state in the process of social welfare system reforms.

The rural working class has historically played a significant role in China’s economic, cultural, and political fabric. As the welfare system undergoes transformation, it is crucial for policymakers to consider the specific needs and challenges faced by migrant workers and take proactive steps to ensure their inclusion and support within the framework of social assistance programs. Potential improvement measures may include supporting businesses to establish more flexible employment systems. Providing migrant workers with more flexible leave options to attend to their family needs, or offering incentives to foster intergenerational living arrangements. Such steps require a pragmatic shift in national and government-level policymaking, recognizing the vital role played by migrant workers as providers for their families and caregivers. Practical support should be provided to alleviate their burdens while simultaneously working toward reducing gender disparities in caregiving responsibilities.

### 5.4 Advantages, limitations and prospects

This study is the first to discuss the economic burden of illness in migrant worker families and also the first to associate this burden with poverty. We employed a multidimensional poverty index that has demonstrated good stability and feasibility in previous studies, applying the A-F method for calculation and decomposition by gender and age. Our research innovatively explores an assessment method for the economic burden of illness based on subjective perception, revealing that the economic burden brought by illness in family members significantly increases the poverty risk among migrant workers, with this impact varying by age and gender.

However, we must acknowledge certain limitations. As this is a cross-sectional study, the temporal sequence of variables cannot be determined, thus the direction of causality remains unclear. Future studies should longitudinally examine these associations. Another limitation stems from the CMDS data, as the survey results on the economic burden of illness are binary (0–1), preventing us from obtaining specific disease information or assessments of the degree of economic burden from the migrant workers’ perspective. Future research should expand and break through from here, combining subjective and objective assessments of the economic burden of illness among migrant workers, as well as personal and family burdens, to view the living conditions, poverty, health, and social security of this group from a more comprehensive perspective.

## 6 Conclusion

This study examines the impact of the economic burden of disease from family members on the poverty of young migrant workers. Expenditure on medical treatment is a significant factor affecting to poverty. Chinese migrant workers not only serve as the primary workforce for urbanization but also the breadwinner of their families. Their ability to withstand risks is easily affected by the healthcare needs of their family members. In contrast to previous studies that objectively calculate the cost of disease expenditure, we explore a subjective perception-based evaluation approach. Our findings reveal the presence of a unique “financial toxicity” among young migrant workers. Based on the China Migrants Dynamic Survey data, it is estimated that young migrant workers in China experience severe multidimensional poverty, with a poverty incidence rate of 30.93%. Young male migrant workers bear a higher economic burden of family member illness (EBFI) than females. However, the risk of multidimensional poverty among young female migrant workers is slightly higher than among males, primarily reflected in the dimensions of income and livelihood. The economic burden of family member’s illness is significantly correlated with the exacerbation of multidimensional poverty among young migrant workers, with females and the 26–35 age group being more susceptible to this adverse impact.

## Data Availability

The original contributions presented in the study are included in the article/supplementary material, further inquiries can be directed to the corresponding authors.

## Footnotes

^1^ https://www.gov.cn/lianbo/bumen/202405/content_6948813.htm
